# Short-term efficacy and safety of CO_2_ laser vaporization for anal intraepithelial neoplasia

**DOI:** 10.3389/fmed.2026.1950769

**Published:** 2026-09-16

**Authors:** Tingting Su, Yi Yu, Fang Chen, Hongwei Zhang, Lu Zhang, Qing Cong, Long Sui, Limei Chen

**Affiliations:** 1 Cervical Diseases Center, Obstetrics and Gynecology Hospital of Fudan University, Yangtze River Delta Integration Demonstration Zone (QingPu), Shanghai, China; 2 Perianal Clinic, Obstetrics and Gynecology Hospital of Fudan University, Shanghai, China; 3 Shanghai Key Laboratory of Female Reproductive Endocrine Related Diseases, Obstetrics and Gynecology Hospital of Fudan University, Shanghai, China

**Keywords:** anal intraepithelial neoplasia, carbon dioxide laser, high-resolution anoscopy, high-risk HPV, laser vaporization

## Abstract

**Objective:**

To evaluate the short-term efficacy, safety, and human papillomavirus (HPV) clearance outcomes of carbon dioxide (CO_2_) laser vaporization in patients with anal intraepithelial neoplasia (AIN), and to identify independent predictors of persistent postoperative HPV infection.

**Methods:**

A retrospective cohort study was conducted on 99 patients who underwent CO_2_ laser ablation for AIN at the Obstetrics and Gynecology Hospital of Fudan University between October 2023 and October 2024. Patients were categorized into four groups based on lesion location (perianal vs. anal canal) and histological grade (low-grade vs. high-grade). Operative parameters, response rates, HPV clearance rates, and complications were systematically analyzed.

**Results:**

Among 99 enrolled patients, 7 were lost to follow-up, leaving 92 cases for final analysis. With a mean follow-up of 9.96 months, the overall 3-month treatment response rate was 85.9% (79/92), and the high-risk HPV clearance rate was 70.7% (65/92). No severe complications such as anal stenosis, incontinence, or mucosal erosion were observed during follow-up. Only 5 cases (5.4%) developed mild perianal redness, which resolved with topical antibiotics. Multivariate logistic regression identified preoperative HPV16/18 infection (OR = 3.62, 95% CI: 1.41–9.28, *P* = 0.007), high-grade lesions (OR = 2.95, 95% CI: 1.13–7.71, *P* = 0.027), and multifocal lesions (OR = 2.43, 95% CI: 1.02–5.80, *P* = 0.044) as independent risk factors for persistent postoperative HPV infection.

**Conclusion:**

CO_2_ laser vaporization demonstrates promising short-term efficacy and a favorable safety profile for treating AIN, and can be considered as an effective short-term local ablative option, though long-term prospective validation is needed. Rigorous surveillance for persistent HPV16/18 infection is warranted, and extended follow-up is required to assess long-term recurrence dynamics.

## Introduction

Anal intraepithelial neoplasia (AIN) comprises a spectrum of intraepithelial lesions strongly associated with persistent high-risk human papillomavirus (HPV) infection. It is considered a precursor to anal squamous cell carcinoma (ASCC). Although anal cancer accounts for a relatively small proportion of global malignancies, its incidence has been rising steadily. In the United States, the average annual increase reached 2.7% between 2001 and 2015. In several high-income countries, anal cancer is projected to surpass cervical cancer as the most common HPV-related malignancy among middle-aged and elderly women (1). The American Cancer Society estimates approximately 10,000 new anal cancer cases in the United States in 2024, with global annual new cases already exceeding 50,000 (2). These statistics underscore the urgent need for early detection and intervention for AIN.

High-resolution anoscopy (HRA)-guided targeted biopsy is widely accepted as the diagnostic gold standard for AIN (3). However, a universally standardized therapeutic protocol remains lacking. The 2023 Chinese expert consensus on the diagnosis and management of AIN emphasizes that the primary therapeutic goals are to alleviate clinical symptoms and prevent progression to invasive carcinoma (4). Currently available local ablative modalities—including surgical excision, electrocautery, and cryotherapy—are associated with notable limitations, such as high recurrence rates, delayed wound healing, and potential compromise of anal sphincter function (5).

Carbon dioxide (CO_2_) laser vaporization offers several distinct advantages. It is characterized by precise tissue ablation, simultaneous hemostasis, and a minimally invasive profile. This technique has demonstrated favorable efficacy and safety in the management of HPV-related precancerous lesions of the cervix, vagina, and vulva (6). In recent years, this technology has been increasingly applied to AIN. Both prospective and retrospective studies have shown that CO_2_ laser achieves effective clearance of anal high-grade squamous intraepithelial lesions (HSIL), with an overall response rate of 70.8% (complete response: 50%; partial response: 20.8%). Furthermore, 69.4% of patients experienced negligible postoperative discomfort, indicating excellent tolerability (7). Compared with conventional electrocautery, CO_2_ laser surgery provides greater precision and less lateral thermal spread, which may improve preservation of adjacent normal mucosa. This substantially reduces the risk of sphincter dysfunction—a critical advantage for preserving anal physiological integrity.

Given these potential benefits, the present study retrospectively analyzed single-center clinical data on CO_2_ laser vaporization for AIN. We aimed to systematically evaluate its short-term efficacy, safety, and HPV clearance outcomes. We also sought to provide evidence-based references for optimizing minimally invasive treatment algorithms and individualized follow-up strategies.

## Methods

### Study subjects

This retrospective study included patients diagnosed with anal intraepithelial neoplasia (AIN) via high-resolution anoscopy (HRA)-guided biopsy at the Cervical Clinic, Vulvar Clinic, and Perianal Clinic of the Obstetrics and Gynecology Hospital of Fudan University between October 2023 and October 2024. This retrospective study was approved by the Institutional Review Board of the Obstetrics and Gynecology Hospital of Fudan University (Approval No. KYY2024-22), which waived the requirement for informed consent. The study was conducted in accordance with the Declaration of Helsinki.

Inclusion criteria were: (1) histologically confirmed anal low-grade squamous intraepithelial lesion (LSIL) or high-grade squamous intraepithelial lesion (HSIL); (2) consent to undergo CO_2_ laser vaporization; (3) complete clinical and follow-up data; and (4) follow-up duration of at least 3 months.

Exclusion criteria were: (1) immunosuppressive status, such as HIV infection, active syphilis, or long-term use of immunosuppressive agents (≥3 months); (2) pregnancy; (3) previous history of anal malignancy or pelvic radiotherapy; and (4) refusal to participate in follow-up or lost to follow-up.

A total of 99 patients (29 males and 70 females) were enrolled, with a mean age of 46.5 ± 11.4 years (range: 22–63 years). Based on lesion location (perianal vs. anal canal) and histological grade (LSIL vs. HSIL), patients were categorized into four groups. Four patients with concurrent involvement of both perianal and anal canal lesions were assigned to their respective groups without duplicate counting.

### Surgical procedure

A JC25 ultrapulse CO_2_ laser system (Juehua Laser Technology Development Co., Ltd., Shanghai, China) was used, with a wavelength of 10.6 μm, spot diameter of 0.8 mm, output power of 12–15 W, and continuous output mode. The treatment room was equipped with a smoke evacuation system.

#### Preoperative preparation

Patients were instructed to consume a low-residue diet one day before surgery and to empty the bowels prior to treatment. Routine screening for syphilis, HIV, hepatitis B, and hepatitis C was performed to exclude acute infections.

#### Anesthesia and positioning

Patients were placed in the left lateral decubitus position with the hips and knees flexed. The perianal skin was cleansed and disinfected. For perianal lesions, local infiltration anesthesia was achieved with 5 mL of 2.0% lidocaine hydrochloride. For anal canal lesions, lidocaine injection was instilled into the anal canal for topical anesthesia. Neither regional nor general anesthesia was required.

#### Laser vaporization procedure

The procedure comprised the following steps. First, a cotton ball soaked in 5% acetic acid was applied to the perianal and anal canal region for 2 min, and the extent of acetowhitening was evaluated under HRA to define the surgical margins. Second, the handpiece was aimed at the lesion, with scanning performed from the periphery to the center and from superficial to deeper layers. The vaporization depth was 1–2 mm for LSIL and 2–3 mm for HSIL, until the wound bed exhibited a uniform yellowish-white coagulative appearance with no macroscopically visible residual lesion. For anal canal lesions, ablation was performed in either a single session or fractionated sessions to achieve complete eradication. For perianal vegetations or lesions, vaporization was extended 2 mm outward from the lesion margin and confined to the superficial dermis to avoid thermal injury to deeper tissues. Third, compression hemostasis was applied using cotton swabs or gauze. Fourth, the wound was disinfected after the procedure. For perianal sites, topical antibiotic ointment was applied to prevent infection, whereas no special treatment was required for anal canal lesions, given the superficial nature of the wounds and their tendency to heal by secondary intention. Postoperatively, patients were instructed to keep the perianal area clean and dry. Topical mupirocin ointment was applied to perianal wounds for 5–7 days to prevent infection. Simple analgesics (e.g., paracetamol) were recommended as needed for pain relief. Stool softeners were prescribed to reduce discomfort during defecation.

#### Fractionated treatment strategy

For lesions with a maximum diameter exceeding 2 cm or involving three or more quadrants, a “zonal, staged” strategy was adopted, with each session ablating no more than 1.5 cm^2^ of the lesion area, at intervals of 4 weeks, until complete eradication was achieved. The operative time was recorded for all patients from the initiation of anesthesia to the completion of hemostasis.

#### Auxiliary technique

For large perianal condylomatous lesions, preoperative electrocautery pretreatment may be applied to coagulate the tissue and reduce intraoperative bleeding, followed by gentle removal of the eschar with a cotton swab prior to laser vaporization. Energy parameters should be carefully controlled during the procedure to avoid thermal injury to surrounding tissues.

### Outcome measures

#### Primary outcome measures

The primary outcomes were: (1) the treatment response rate at 3 months postoperatively, defined as the absence of any visible lesion on high-resolution anoscopy (HRA) or negative histopathology on targeted biopsy; and (2) the high-risk HPV clearance rate at 3 months postoperatively, defined as negative results on HPV genotyping and quantitative testing.

HPV genotyping and quantitative testing were performed using the BioPerfectus Multiplex Real-Time (BMRT) HPV Genotyping Kit (fluorescence PCR method) (Jiangsu BioPerfectus Technologies Co., Ltd., China). This kit is based on a multiplex real-time PCR platform and uses separate well-based genotyping with closed-tube detection of 21 HPV subtypes, enabling simultaneous HPV genotyping and viral load quantification. The 21 HPV subtypes detected include 18 high-risk types (HPV16, 18, 31, 33, 35, 39, 45, 51, 52, 53, 56, 58, 59, 66, 68, 26, 73, and 82) and 3 low-risk types (HPV6, 11, and 81). The assay employs the human TOP3 single-copy gene to normalize the number of collected cells, and viral load is reported as HPV copies per 10,000 cells. Negative results were defined according to the manufacturer's cutoff values. For patients receiving fractionated treatment, the 3-month postoperative evaluation was calculated from the date of the final treatment session.

#### Secondary outcome measures

The secondary outcomes included: (1) operative time, measured from the initiation of anesthesia to the completion of hemostasis; (2) postoperative pain intensity, assessed using a visual analog scale (VAS) ranging from 0 (no pain) to 10 (worst possible pain); (3) wound healing status at 1 month postoperatively (for fractionated patients, assessed at 1 month after the final session), defined as complete epithelialization without signs of infection, dehiscence, or persistent exudate; and (4) procedure-related complications, including but not limited to anal stenosis, incontinence, infection, bleeding, and mucosal erosion.

#### Risk factor analysis

Candidate variables for risk factor analysis included age, sex, lesion location (perianal vs. anal canal), pathological grade (LSIL vs. HSIL), number of lesions (single vs. multifocal), preoperative HPV16/18 infection (yes/no), baseline viral load (expressed as HPV copies/10,000 cells, as quantitatively determined by the BMRT assay), smoking history (yes/no), and presence of synchronous multifocal lower genital tract lesions. Candidate variables were first screened by univariate analysis. Variables with *P* < 0.05 were then entered into the multivariate model. The detailed statistical methods are described in the Statistical Analysis section below.

#### Follow-up protocol

Follow-up visits were scheduled at 1, 3, 6, and 12 months postoperatively. At 1 month, wound healing status was assessed (for patients receiving fractionated treatment, assessed at 1 month after the final session). At 3 months, all patients underwent anal cytology, high-risk HPV genotyping and quantitative testing, and HRA examination; targeted biopsy was performed for any HRA-suspicious areas. Thereafter, patients were followed up every 6 months with anal cytology and HPV testing; HRA was repeated if either test was abnormal. Clinical assessments, including VAS pain scores and recording of adverse events, were performed at each visit. The minimum follow-up duration was 3 months, with a planned total follow-up of at least 2 years. Loss to follow-up was defined as two consecutive missed appointments with inability to contact the patient by telephone. Patients who withdrew consent were excluded from the final analysis but were not considered lost to follow-up.

#### Statistical analysis

Statistical analyses were performed using SPSS version 26.0 (IBM Corp., Armonk, NY, USA). Normality of continuous variables was assessed using the Shapiro–Wilk test. Normally distributed continuous data were expressed as mean ± standard deviation (SD) and compared using the independent samples *t*-test (for two groups) or one-way analysis of variance (ANOVA) (for four groups). Non-normally distributed continuous data were expressed as median with interquartile range (IQR) and compared using the Mann–Whitney *U*-test (for two groups) or Kruskal–Wallis test (for four groups). Categorical variables were expressed as frequencies (percentages) and compared using the *χ*^2^ test or Fisher's exact test, as appropriate. Multivariate logistic regression analysis (forward likelihood ratio method) was used to identify independent risk factors for persistent postoperative HPV infection. A two-sided *P*-value < 0.05 was considered statistically significant.

## Results

### Baseline characteristics

All 99 patients included in this study tested negative for syphilis and HIV before surgery, and none had immunosuppressive conditions. The cohort comprised 29 males and 70 females, with a mean age of 46.5 ± 11.4 years (range: 22–63 years). Based on lesion location and pathological grade, patients were categorized into four groups: perianal LSIL (*n* = 42), perianal HSIL (*n* = 18), anal canal LSIL (*n* = 28), and anal canal HSIL (*n* = 15). For patients with mixed LSIL and HSIL lesions, classification was based on the highest pathological grade detected on biopsy. Four patients had concurrent perianal and anal canal lesions and were assigned to their respective groups without duplicate counting.

Asymptomatic patients accounted for 70.7% (70/99), while 29.3% (29/99) were symptomatic. The most common presentations were anal pruritus (48.3%, 14/29), anal vegetations (41.4%, 12/29), and anal pain (10.3%, 3/29). Age differed significantly among the four groups (*F* = 18.36, *P* < 0.001), with HSIL groups being older than LSIL groups. However, no significant differences were observed among the four groups in terms of sex (*χ*^2^ = 0.97, *P* = 0.806), symptoms (*χ*^2^ = 6.06, *P* = 0.108), or preoperative cytology (*χ*^2^ = 2.44, *P* = 0.486) (Table 1). Among the 70 female patients, 28 (40.0%) had concurrent multifocal lower genital tract lesions, including vulvar lesions in 17 cases, vaginal lesions in 6 cases, and cervical lesions in 10 cases. Five patients had involvement of three or more sites simultaneously.

**Table 1:** Baseline characteristics of patients with anal intraepithelial neoplasia before treatment [*x* ± *s* or *n*(%)].

Characteristic	P-LSIL (*n* = 42)	P-HSIL (*n* = 18)	A-LSIL (*n* = 28)	A-HSIL(*n* = 15)	*p*-value
Age (years, x ± s)^a^	35.4 ± 6.0	46.8 ± 5.9	35.6 ± 4.8	43.7 ± 7.7	<0.001
Sex					
Male	10 (23.8%)	6 (33.3%)	9 (32.1%)	5 (33.3%)	0.806
Female	32 (76.2%)	12 (66.7%)	19 (67.9%)	10 (66.7%)	
Symptoms					
Asymptomatic	28 (66.7%)	10 (55.6%)	24 (85.7%)	12 (80.0%)	0.108
Symptomatic	14 (33.3%)	8 (44.4%)	4 (14.3%)	3 (20.0%)	
Preoperative cytology^b^					
NILM	16 (38.1%)	3 (16.7%)	9 (32.1%)	2 (13.3%)	
ASCUS	14 (33.3%)	3 (16.7%)	6 (21.4%)	4 (26.7%)	
LSIL	9 (21.4%)	6 (33.3%)	11 (39.3%)	4 (26.7%)	
ASC-H	2 (4.8%)	4 (22.2%)	2 (7.1%)	3 (20.0%)	
HSIL	1 (2.4%)	2 (11.1%)	0 (0.0%)	2 (13.3%)	
Preoperative HPV status					
HPV16 infection	22 (52.4%)	16 (88.9%)	15 (53.6%)	15 (100%)	<0.001
Other high-risk HPV infection	7 (16.7%)	5 (27.8%)	5 (17.9%)	4 (26.7%)	0.697
Low-risk HPV infection	17 (40.5%)	0 (0.0%)	11 (39.3%)	0 (0.0%)	<0.001

a Data are presented as mean ± SD or *n* (%), unless otherwise specified.

b Preoperative cytological diagnoses were classified according to the Bethesda System (TBS) 2014.

NILM, negative for intraepithelial lesion or malignancy; ASCUS, atypical squamous cells of undetermined significance; LSIL, low-grade squamous intraepithelial lesion; ASC-H, atypical squamous cells cannot exclude high-grade lesion; HSIL, high-grade squamous intraepithelial lesion; P-LSIL, perianal LSIL; P-HSIL, perianal HSIL; A-LSIL, anal canal LSIL; A-HSIL, anal canal HSIL.

### Preoperative cytology and HPV infection

With increasing lesion grade, both abnormal cytology results and high-risk HPV positivity rates showed an upward trend. In the LSIL group, abnormal cytology rates were 61.9% (26/42) for perianal lesions and 67.9% (19/28) for anal canal lesions, with high-risk HPV positivity rates of 54.8% (23/42) and 60.7% (17/28), respectively. In the HSIL group, abnormal cytology rates were 83.3% (15/18) for perianal lesions and 86.7% (13/15) for anal canal lesions, with high-risk HPV positivity rates of 100% in both locations (18/18 for perianal, 15/15 for anal canal). The anal canal HSIL group had the highest HPV16 infection rate (100%, 15/15) and the highest proportion of mixed infections (33.3%, 5/15).

When perianal and anal canal LSIL were combined into the LSIL group (*n* = 70) and perianal and anal canal HSIL into the HSIL group (*n* = 33), the median viral load in the HSIL group was significantly higher than that in the LSIL group (206.3 vs. 89.7 copies/10,000 cells, *Z* = −4.52, *P* < 0.001) (Table 1).

### Operative outcomes

Ninety-three patients underwent a single session of laser vaporization, while 6 patients (4 perianal, 2 anal canal) received fractionated treatment due to large lesion size (maximum diameter > 2 cm) or multifocal lesions, with a mean of 1.9 sessions per patient. The mean operative time was 3.2 ± 0.8 min, intraoperative blood loss was 2.1 ± 1.3 mL, and the VAS pain score at 24 h postoperatively was 2.1 ± 0.9, with no need for additional analgesia. No immediate complications such as perforation, major bleeding, or infection occurred postoperatively.

### Follow-up and treatment outcomes

Seven patients were lost to follow-up, leaving 92 patients available for analysis, with a mean follow-up duration of 9.96 ± 2.79 months. The overall 3-month treatment response rate was 85.9% (79/92), with response rates of 76.2% (32/42) for perianal LSIL, 83.3% (15/18) for perianal HSIL, 67.9% (19/28) for anal canal LSIL, and 86.7% (13/15) for anal canal HSIL. No significant difference in response rates was observed among the four groups (*χ*^2^ = 2.509, *P* = 0.474). One patient with perianal HSIL still had HSIL on pathology at 3-month follow-up and achieved complete remission after a second laser treatment (Table 2).

**Table 2:** Comparison of lesion changes and high-risk HPV clearance at 3-month follow-up after treatment among patients with anal lesions (%).

Parameter (*n*)	P-LSIL(*n* = 42)	P-HSIL (*n* = 18)	A-LSIL (*n* = 28)	A-HSIL(*n* = 15)	*χ*^2^ value	*p*-value
Lesion change						
Complete remission	32 (76.2%)	15 (83.3%)	19 (67.9%)	13 (86.7%)	2.509	0.474
Dual negative (HR-HPV and cytology)	30 (71.4%)	14 (77.8%)	19 (67.9%)	11 (73.3%)	0.789	0.852
LSIL	8 (19.0%)	1 (5.6%)	6 (21.4%)	1 (6.7%)	3.400	0.334
HSIL	0 (0.0%)	1 (5.6%)	0 (0.0%)	0 (0.0%)	—	0.209
HR-HPV test result						
HR-HPV clearance	32 (76.2%)	15 (83.3%)	19 (67.9%)	12 (80.0%)	1.664	0.645
HPV16/18 positive	5 (11.9%)	1 (5.6%)	1 (3.6%)	1 (6.7%)	1.732	0.630
Non-HPV16/18 positive	3 (7.1%)	1 (5.6%)	5 (17.9%)	1 (6.7%)	3.219	0.359
HR-HPV infection status						
Single infection clearance	28 (66.7%)	12 (66.7%)	16 (57.1%)	9 (60.0%)	0.891	0.828
Multiple infection clearance	4 (9.5%)	3 (16.7%)	3 (10.7%)	3 (20.0%)	1.479	0.687

LSIL, low-grade squamous intraepithelial lesion; HSIL, high-grade squamous intraepithelial lesion; HR-HPV, high-risk human papillomavirus; P-LSIL, perianal LSIL; P-HSIL, perianal HSIL; A-LSIL, anal canal LSIL; A-HSIL, anal canal HSIL.

In terms of safety, 5 patients with perianal lesions developed mild redness at 7 days postoperatively (a normal postoperative reaction), which resolved with topical application of mupirocin ointment without infection. No complications such as anal stenosis, incontinence, or local erosion occurred during follow-up, and no lesion progression was observed.

Representative images of anal canal condylomata acuminata and HSIL before and 3 months after CO_2_ laser vaporization are shown in Figures 1, 2, respectively.

**Figure 1:**
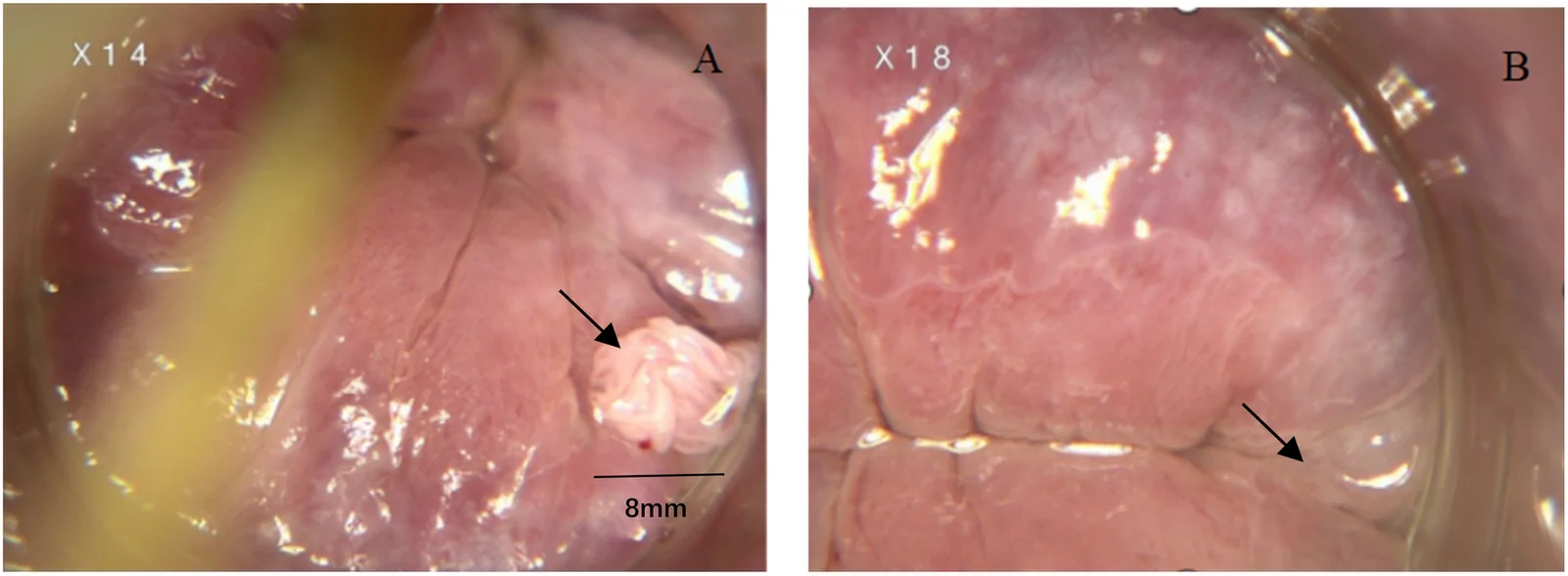
Anal canal condylomata acuminata. **(A)** Pre-treatment HRA image showing multiple exophytic verrucous lesions with acetowhite epithelium in the anal canal. **(B)** At 3 months post-CO_2_ laser vaporization, showing complete clearance of the lesions with well-epithelialized mucosa and no evidence of residual disease.

**Figure 2:**
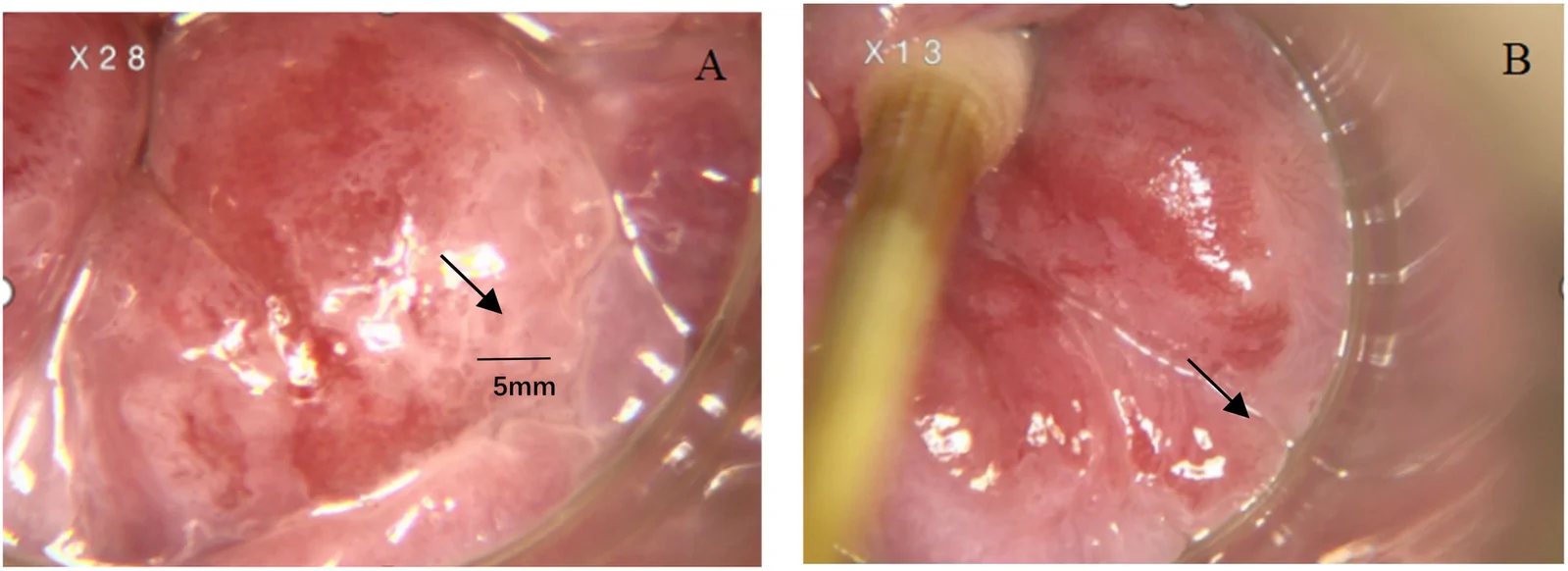
High-grade squamous intraepithelial lesion (HSIL) of the anal canal. **(A)** Pre-treatment HRA image showing a well-demarcated acetowhite area with coarse punctation and atypical vessels, consistent with HSIL. **(B)** At 3 months post-CO_2_ laser vaporization, showing complete regression of the acetowhite epithelium with restoration of normal vascular patterns and no residual lesion. Lesion sizes (indicated on the images) were estimated based on clinical documentation.

### HPV clearance

The overall high-risk HPV clearance rate at 3 months postoperatively was 70.7% (65/92). In the anal LSIL group, the HPV infection rate decreased from 98.5% (67/68) preoperatively to 28.6% (18/63) at 3 months postoperatively. In the anal HSIL group, the HPV infection rate decreased from 100% (31/31) to 31.0% (9/29). Both differences were statistically significant (McNemar *χ*^2^ = 49.0 and 22.0, respectively, both *P* < 0.001) (Table 3).

**Table 3:** HPV infection rates before and after laser treatment (%).

Parameter	Perianal and anal canal LSIL	Perianal and anal canal HSIL
Pre-treatment HPV infection	98.5% (67/68)	100% (31/31)
Post-treatment HPV infection	28.6% (18/63)	31.0% (9/29)
*χ* ^2^	49.0	22.0
P-value	<0.001	<0.001

In patients with persistent HPV infection (still positive at 3 months postoperatively), viral load also decreased compared with preoperative levels. The mean viral load was 32.5 ± 14.3 copies/10,000 cells preoperatively and 5.2 ± 3.5 copies/10,000 cells postoperatively. However, because the HPV genotypes detected postoperatively differed from those identified preoperatively in a substantial proportion of patients, viral load data were not included in the standardized statistical analysis in this study.

The observed genotype discordance between pre- and post-treatment samples raises the question of whether postoperative genotype shifts reflect newly acquired HPV infections or the unmasking of latent, previously undetectable infections. However, our current data do not allow definitive differentiation between these two possibilities, as this would require longitudinal genotyping spanning multiple time points before, during, and after treatment—data that were not available in this retrospective study. Furthermore, the limited number of patients with concordant pre- and post-treatment genotypes precludes meaningful subgroup analysis. These results are therefore presented for reference only and require further validation in prospective studies with comprehensive longitudinal sampling.

### Risk factors for persistent postoperative HPV infection

Univariate analysis revealed that preoperative HPV16/18 infection, lesion grade (HSIL), lesion number (multifocal), and viral load >100 copies/10,000 cells (using the median viral load of 100 copies/10,000 cells as the cutoff value) were associated with persistent postoperative HPV infection (all *P* < 0.05). These variables were subsequently entered into a multivariate logistic regression model using the forward likelihood ratio method.

The results showed that preoperative HPV16/18 infection (OR = 3.62, 95% CI: 1.41–9.28, *P* = 0.007), HSIL (OR = 2.95, 95% CI: 1.13–7.71, *P* = 0.027), and multifocal lesions (OR = 2.43, 95% CI: 1.02–5.80, *P* = 0.044) were independent risk factors for persistent postoperative HPV infection (Table 4). Although a baseline viral load >100 copies/10,000 cells was significant in univariate analysis, it did not remain in the final multivariate model.

**Table 4:** Multivariate logistic regression analysis of factors associated with persistent postoperative HPV infection.

Variables	*β*	SE	Wald *χ*^2^	OR	95% CI	*P*-value
Preoperative HPV16/18 infection (yes vs. no)	1.286	0.481	7.151	3.62	1.41–9.28	0.007
Lesion grade (HSIL vs. LSIL)	1.082	0.490	4.879	2.95	1.13–7.71	0.027
Lesion number (multifocal vs. unifocal)	0.888	0.443	4.018	2.43	1.02–5.80	0.044

Dependent variable: persistent postoperative HPV infection (0 = clearance, 1 = persistence). Variables with *P* < 0.05 in univariate analysis were entered into the multivariate model using forward likelihood ratio selection. Reference categories: no HPV16/18 infection for preoperative HPV16/18 infection; LSIL for lesion grade; unifocal for lesion number. CI, confidence interval; OR, odds ratio; SE, standard error.

## Discussion

In this study, we systematically evaluated the short-term efficacy, safety, and HPV clearance outcomes of CO_2_ laser vaporization for AIN, and explored the risk factors for persistent postoperative HPV infection. Our results demonstrated an overall 3-month treatment response rate of 85.9%, a high-risk HPV clearance rate of 70.7%, and no severe complications, confirming the favorable short-term efficacy and safety profile of this technique in the treatment of AIN. Multivariate analysis further identified preoperative HPV16/18 infection, HSIL, and multifocal lesions as independent risk factors for persistent postoperative HPV infection, providing evidence-based support for individualized follow-up strategies in clinical practice. These findings underscore the importance of both technical efficacy and patient-specific risk stratification in optimizing AIN management.

### Epidemiology of AIN and implications for screening

AIN predominantly occurs in individuals with persistent HR-HPV infection, particularly HIV-positive individuals, men who have sex with men, and women with multifocal HPV-related lesions (8–11). In the present study, 70.7% of patients were asymptomatic, highlighting the insidious nature of AIN and underscoring the importance of anal cytology combined with HRA screening in high-risk populations. Furthermore, 28.6% (28/70) of female patients in this cohort had concurrent multifocal lower genital tract lesions, showing a significant association with vulvar, vaginal, and cervical lesions. This finding is consistent with the well-documented phenomenon of multifocal HPV-related neoplasia across multiple anatomical sites (12). We therefore recommend that women with multifocal lower genital tract lesions be prioritized for AIN screening, and that a “one-stop” multidisciplinary management model for multiple anatomical sites be explored to achieve comprehensive prevention and control of HPV-related diseases. It is important to emphasize that our findings, derived from an immunocompetent cohort, cannot be directly extrapolated to HIV-positive individuals, who constitute a high-risk population with potentially different treatment durability and recurrence patterns. Future studies specifically addressing HIV-positive cohorts are warranted.

Although our study excluded HIV-positive individuals to avoid confounding by immunosuppression, the epidemiological features of AIN in immunocompetent populations share certain similarities with those in HIV-positive cohorts, including the predominance of asymptomatic cases and the association with HR-HPV infection.

### Technical advantages and operative considerations of CO_2_ laser

The CO_2_ laser, with a wavelength of 10.6 μm, has a high water absorption coefficient and a penetration depth of only 0.1–0.2 mm, enabling precise vaporization of lesional tissue while maximally preserving normal mucosa. This physical property confers a unique advantage for the treatment of anal lesions (13, 14). In the present study, the mean operative time was only 3.2 min, intraoperative bleeding was minimal, the VAS pain score at 24 h postoperatively was 2.1 ± 0.9, wound healing was rapid, and no severe complications such as anal stenosis or incontinence occurred. These findings are consistent with the safety profile reported for CO_2_ laser treatment of vulvovaginal HSIL (15). Collectively, these results indicate that CO_2_ laser vaporization is a minimally invasive, safe, and highly effective modality for the treatment of AIN.

Given the complex anatomy of the anal canal, precise localization under HRA guidance is essential during the procedure. Based on our institutional experience, key operative points include: (1) acetic acid application for ≥ 90 s to ensure adequate lesion visualization; (2) use of a 30° angled scope for distal anal canal lesions to improve visualization; (3) establishment of a “clock-face” mapping system for multifocal lesions to avoid omission; and (4) real-time smoke evacuation during the procedure to minimize viral aerosol exposure. To date, reports on CO_2_ laser therapy specifically for AIN in China remain limited, and the present study is one of the first to systematically validate the feasibility of this technique in a Chinese population, offering a novel minimally invasive option for the treatment of AIN.

### CO_2_ Laser ablation versus topical medical therapies: comparative evidence and clinical implications

Current AIN treatment strategies primarily include local ablative therapies (CO_2_ laser, electrocautery, infrared coagulation) and topical agents (imiquimod, 5-fluorouracil, cidofovir). Direct comparisons between these modalities are challenging due to heterogeneous study designs and outcome definitions. Nevertheless, existing evidence offers valuable clinical insights.

Regarding lesion clearance, the 3-month treatment response rate of 85.9% with CO_2_ laser in our study compares favorably with reported outcomes of topical therapies. A systematic review reported complete response rates of 14%–79% for imiquimod, 9%–86% for 5-fluorouracil, 15%–59% for cidofovir, and 28%–72% for trichloroacetic acid (16). Notably, those studies predominantly enrolled HIV-positive MSM, whereas our immunocompetent cohort may partly explain the higher response rates observed.

In the only randomized trial directly comparing modalities, electrocautery achieved a higher complete response rate (39%) than imiquimod (24%) or 5-fluorouracil (17%) (16), suggesting that ablative therapy may offer superior initial clearance in immunocompromised populations.

Regarding viral clearance, our study demonstrated an overall high-risk HPV clearance rate of 70.7%. Topical imiquimod has been shown to reduce lesional HPV DNA load in complete responders (17), though non-standardized assays and limited sample sizes preclude definitive conclusions.

Side effects and adherence favor CO_2_ laser due to its single-session nature and low adverse event rate (5.4% in our cohort). Topical therapies require prolonged self-administration and are associated with higher local adverse events (imiquimod >90%, 5-FU approximately 85%) (16, 17). Although most are mild to moderate, 9%–21% of patients discontinue treatment due to severe side effects (16).

Recurrence risk remains high across all modalities, particularly in HIV-positive populations (16–18). With a median follow-up of 9.96 months, our study cannot yet provide definitive long-term recurrence estimates.

Given the complementary profiles of ablative and topical therapies—immediate lesion eradication vs. sustained immunological effect—a combined or sequential approach (initial CO_2_ laser ablation followed by topical immunomodulation) could theoretically offer superior outcomes for high-risk cohorts by combining immediate lesion clearance with sustained antiviral immune activation, though this hypothesis requires prospective validation (16).

In summary, CO_2_ laser ablation offers advantages in short-term lesion clearance and patient acceptability, while topical therapies serve as alternatives for patients unsuitable for or averse to invasive procedures. For high-risk patients, future studies should optimize combined or sequential strategies.

Despite the encouraging short-term outcomes described above, a substantial proportion of patients (29.3%) remained HR-HPV positive postoperatively, underscoring the need for optimized surveillance and management strategies for persistent infection.

### Persistent HPV infection and follow-up strategy

Postoperatively, 29.3% of patients remained HR-HPV positive, which may be attributable to viral integration status, immune evasion mechanisms, and latent HPV infection in basal cells of the lesion (19, 20). Multivariate analysis further confirmed that preoperative HPV16/18 infection, HSIL, and multifocal lesions were independent risk factors for persistent postoperative HPV infection. Among these, HPV16/18 infection had the highest OR value (OR = 3.62), suggesting that this genotype exerts the most significant negative impact on viral clearance after laser treatment, which may be related to the stronger transforming capacity and higher integration propensity of HPV16/18. Based on these findings, we recommend combined anal cytology and HPV testing at 3, 6, and 12 months postoperatively, with HRA repeated every 6 months for patients with persistent positivity. For patients with persistent HPV16/18 infection or multifocal lesions, topical immunomodulatory agents (as discussed in the preceding section) or systemic immunomodulatory therapy may be considered to reduce the risk of recurrence (21).

### Limitations and future directions

Several limitations of this study should be considered when interpreting the results.

First, this was a retrospective study, which may have introduced selection bias, and the data quality was dependent on the completeness and accuracy of medical records. Second, this was a single-center study, with all patients enrolled from a single institution, limiting the generalizability and external validity of the findings. Our conclusions warrant validation in multicenter studies. Third, the sample size was relatively limited (99 patients), with particularly small subgroup sizes (e.g., only 15 patients with anal canal HSIL), which may have reduced the statistical power of the multivariate analysis and potentially resulted in failure to detect certain risk factors.

Fourth, the follow-up duration was relatively short (mean 9.96 months), allowing evaluation of only short-term outcomes. Extended follow-up of at least 3 years is therefore warranted to definitively assess long-term recurrence and safety. Fifth, this study excluded HIV-positive patients, who represent a high-risk population for AIN. The patterns of HPV clearance and recurrence in HIV-positive individuals may differ substantially from those in immunocompetent individuals; therefore, whether our findings can be generalized to HIV-positive populations remains unclear. Sixth, the assessment of treatment response partly relied on HRA findings rather than histological biopsy in all cases, which may have introduced false-negative results and led to potential overestimation of the response rate. Seventh, in the analysis of viral load, the HPV genotypes detected postoperatively differed from those identified preoperatively in a substantial proportion of patients. This genotype discordance precluded standardized statistical analysis of viral load changes and also limited our ability to distinguish between newly acquired infections and the unmasking of latent infections that were present at sub-detectable levels before treatment. Distinguishing between these possibilities would require longitudinal genotyping data spanning multiple time points, which were not available in this retrospective study. Consequently, our viral load findings are presented for reference only and require further validation.

Future research directions include: (1) prospective, multicenter, large-sample studies to validate the long-term efficacy of CO_2_ laser; (2) exploration of photodynamic therapy combined with laser ablation as a synergistic strategy (22); (3) integration of HPV vaccination as a supplementary measure in postoperative management, particularly recommending post-operative nine-valent HPV vaccination for unvaccinated HSIL patients (23); and (4) comparative studies including HIV-positive populations to clarify the applicability of this technique across different immune status groups.

## Conclusions

In summary, CO_2_ laser vaporization is a safe, effective, and minimally invasive treatment for AIN, with a high short-term treatment response rate and ease of operation, making it a promising short-term treatment option. However, attention should be paid to the risks of persistent HPV16/18 infection and long-term recurrence; individualized follow-up strategies should be implemented, and large-scale, multicenter studies with extended follow-up are warranted to further validate the long-term efficacy of this approach.

## Data Availability

The raw data supporting the conclusions of this article will be made available by the authors, without undue reservation.
